# Long non-coding RNA SNHG25 promotes epithelial ovarian cancer progression by up-regulating COMP

**DOI:** 10.7150/jca.47344

**Published:** 2021-01-16

**Authors:** Yinglei Liu, Boqun Xu, Manhua Liu, Haifeng Qiao, Siming Zhang, Junjun Qiu, Xiaoyan Ying

**Affiliations:** 1 Department of Obstetrics and Gynecology, the Second Affiliated Hospital of Nanjing Medical University, 262 Zhongshan North Road, Nanjing, 210000, China.; 2 Department of Obstetrics and Gynecology, the Second Affiliated Hospital of Nantong University, 6 Haierxiang North Road, Nantong, 226000, China.; 3 Department of Gynecology, Obstetrics and Gynecology Hospital, Fudan University, 419 Fangxie Road, Shanghai, 200011, China.; 4 Shanghai Key Laboratory of Female Reproductive Endocrine-Related Diseases, 413 Zhaozhou Road, Shanghai, 200011, China.

**Keywords:** lncRNA SNHG25, epithelial ovarian cancer, proliferation, metastasis, COMP

## Abstract

Long non-coding RNAs (lncRNAs) play a pivotal role in the genesis and development of cancer. The role and molecular mechanisms of SNHG25 in epithelial ovarian cancer (EOC) have not been investigated. In the present study, we showed that SNHG25 expression was up-regulated in EOC tissues relative to normal ovarian tissues. *In vitro*, functional experiments demonstrated that high expression of SNHG25 promoted proliferation, migration and invasion, and decreased apoptosis, in ovarian cancer cell lines. *In vivo*, downregulation of SNHG25 inhibited the growth (tumor volume) of subcutaneous xenografts in nude mice. High-throughput sequencing and western blot analysis showed a significant decrease in the expression of COMP mRNA and protein in SNHG25 knockdown compared to control ovarian cancer cells. These data suggest that SNHG25 promotes EOC progression by regulating COMP, serving as a potential biomarker for EOC.

## Introduction

Epithelial ovarian cancer (EOC) is a leading cause of cancer-related mortality 1. Most patients present with advanced disease, when the 5-year overall survival rate is < 30% 2, 3. Diagnosis of early stage EOC is challenging as the disease causes few specific symptoms when it is localized to the ovary 4, 5. The majority of patients with ovarian cancer respond to surgery and chemotherapy; however, recurrence rates are high and prognosis is poor, especially for patients with advanced disease 6. Further understanding of the molecular mechanisms underlying EOC tumor biology is required to develop novel targeted treatment strategies 7.

Long non-coding RNAs (lncRNAs) are noncoding transcripts > 200 bp in length 8. LncRNAs regulate protein synthesis and stability 9, influence cell proliferation, differentiation and survival, and play a role in various diseases 10-12. In cancer, lncRNAs may function as oncogenes or tumor suppressor genes and regulate cancer signaling pathways 13. Dysregulation of lncRNAs has been reported in ovarian cancer 14, gastric cancer^15^, osteosarcoma^16^, and breast cancer^17^. Exploration of lncRNA-based signaling pathways will provide insights into the regulation of cancer progression.

Recently, the lncRNA small nucleolar RNA host gene (SNHG) family has been implicated in several cancers. SNHG4 high expression was associated with tumor size and poor prognosis in patients with osteosarcoma 16. SNHG15 was identified as a potential prognostic marker and therapeutic target in hepatocellular carcinoma (HCC) 18. SNHG25, located in 17q23.3, is a novel lncRNA in ovarian cancer retrieved from The Cancer Genome Atlas (TCGA) (https://cancergenome.nih.gov). To the authors' knowledge, the role and molecular mechanisms of SNHG25 in ovarian cancer have not been investigated.

In this study, we conducted a series of *in vitro* and* in vivo* assays to clarify the roles and mechanisms of SNHG25 in EOC progression. Findings suggest that SNHG25 is involved in EOC proliferation and metastasis, indicating that SNHG25 may be a viable biomarker for EOC.

## Materials and methods

### Patients and tissue sample

This study included 30 ovarian cancer tissue samples and 30 normal ovarian tissue samples collected from patients attending the Department of Obstetrics and Gynecology in the Second Affiliated Hospital of Nantong University between January 2017 and December 2018. Ovarian cancer tissue samples were confirmed as EOC by postoperative pathology. No patient received therapy before surgery. Normal ovarian tissue samples were collected from patients with uterine myoma who underwent uterine and ovarian resection. Fresh samples were snap-frozen and stored at -80 °C until use. This study was approved by the Ethical Committee of the Second Affiliated Hospital of Nantong University.

### Cell culture and establishment of stable knockdown cell lines

The normal ovarian surface epithelial cell line (IOSE80) and the ovarian cancer cell lines, SKOV3, A2780, HEY and OVCAR3, were purchased from Procell Life Science & Technology. Cells were cultured in RPMI-1640 medium (Gibco; Thermo Fisher Scientific, Inc.) supplemented with 10% fetal bovine serum (FBS; Gibco; Thermo Fisher Scientific, Inc.) and 1% penicillin/streptomycin (Gibco; Thermo Fisher Scientific, Inc.) at 37 °C in a humidified atmosphere and 5% CO_2_.

For knockdown of SNHG25, A2780 and OVCAR3 cells were transfected with a lentiviral vector encoding shRNA targeting SNHG25 (5'-3': GGATGTCATCGTCCTTGCT) (LV-KD) or an empty vector as a negative control (LV-NC) (Genepharma, Shanghai, China) using polybrene (5.0 µg/mL). 24h after infection, cells were selected with 0.2 mg/mL puromycin. SNHG25 knockdown efficiency was confirmed with quantitative real-time polymerase chain reaction (qRT-PCR).

### RNA extraction and qRT‐PCR

Total RNA was extracted from frozen tissues or cell lines using TRIzol reagent (Life Technologies, USA). RNA concentrations were measured using a NanoDrop ND-2000 spectrophotometer (NanoDrop Technologies, Wilmington, DE). RNA was reverse-transcribed to cDNA using a reverse transcription kit (Takara, Tokyo, Japan). qRT‐PCR was performed using the SYBR qPCR Master Mix (Vazyme, China), according to the manufacturer's instructions. The primer sequences were: SNHG25 forward primer 5′‐GCAGGTTCCGGGAGGTCA‐3′, SNHG25 reverse primer 5′‐CAAACCACTTTATTGACGGGAA‐3′, GAPDH forward primer 5′-AGAAGGCTGGGGCTCATTTG-3′, GAPDH reverse primer 5′‐AGGGGCCATCCACAGTCTTC‐3′, COMP forward primer 5′‐GGAGATGCTTGTGACAGCGATC‐3′, COMP reverse primer 5′‐TGAGTCCTCCTGGGCACTGTTA‐3′.

Parameters were: pre-denaturation at 95 °C for 10 min for 1 cycle, denaturation at 95 °C for 30 s, annealing at 60 °C for 1 min, and extension at 60 °C for 30 s for a total of 40 cycles. The house keeping gene GAPDH (glyceraldehyde-3-phosphate dehydrogenase) was used as an internal control. The fold change in the expression of target genes was calculated with the 2^-ΔΔCT^ method.

### Western blot

Total protein was extracted with RIPA buffer according to standard protocols. Protein concentration was measured with a BCA protein assay kit (Thermo, Waltham, MA, USA). Proteins were electrophoretically separated on 10% SDS-PAGE gels and transferred onto polyvinylidene difluoride membranes. After blocking, membranes were incubated with primary antibody against COMP (Abcam, USA) or GAPDH (Proteintech, Chicago, IL, USA) at 4 °C overnight. Membranes were washed three times with TBST, incubated with the goat anti-rabbit antibody, and target protein bands were detected with an enhanced chemiluminescence kit (Beyotime, Shanghai, China).

### Cell viability assay

Cell proliferation was assessed using the Cell Counting Kit 8 (CCK-8; Medchem Express), according to the manufacturer's instructions. Transfected cells in the logarithmic growth phase were seeded in triplicate in a 96-well plate at 5000 cells/well. Cells were incubated overnight at 37 °C and 5% CO_2_. Cells were cultured with 100 μl of medium and 10 μl of CCK-8 for 1h. Absorbance of each well was measured at 450 nm at 0h, 24h, 48h, 72h, and 96h using a microplate reader (Tecan, Mechelen, Belgium).

### Cell colony formation assays

Cell proliferation was further investigated using the colony formation assay. Transfected cells were seeded in triplicate in medium plates at 3000 cells/plate, incubated for 2 weeks, washed with PBS, fixed with 4% paraformaldehyde for 2h, and stained with crystal violet for 1h. The number of colonies was counted under a microscope.

### Scratch assay

Cell migration was examined using the scratch assay. Transfected cells were seeded in triplicate into two 6-well plates with complete medium and grown to 100% confluence. The confluence plates were scratched using a sterile pipette tip, medium was replaced with serum-free RPMI-1640 medium, and plates were photographed under a microscope (x10) at 0h and 24h. Cell migration was measured by monitoring the width of the scratch over time using Image J software.

### Cell invasion assay

The cell invasion assay was performed using a Transwell chamber (Millipore, Billerica, MA) coated with Matrigel basement membrane matrix (BD Biosciences, Franklin Lakes, NJ, USA). 200μl of 4 x10^4^ transfected cells suspended in serum-free RPMI-1640 medium was transferred to the upper Matrigel chamber. 600 µl RPMI-1640 medium supplemented with 10% FBS was added to the lower chamber. The chamber was incubated at 37˚C for 36 h. Cells that invaded through the 8 μm membrane were fixed and stained with crystal violet. The number of cells in three random regions was counted using inverted microscopy.

### Flow cytometry

For analysis of apoptosis, transfected cells were seeded in 6-well plates, exposed to annexin-V/PI (BD Biopharmingen, NJ, USA), cultured for 24 hours, and washed and resuspended twice in PBS. Cells were analyzed by flow cytometry (FACScan; BD Biosciences, USA).

### RNA- sequencing

Total RNA was extracted from transfected cells using TRizol Reagent (Life Technologies, USA). Sequencing libraries were generated using the NEBNext® UltraTM RNA Library Prep Kit for Illumina® (NEB, USA) according to the manufacturer's instructions. Index codes were used to attribute sequences to each sample. Clustering of the index-coded samples was performed on a cBot Cluster Generation System using TruSeq PE Cluster Kit v3-cBot-HS (Illumia), according to the manufacturer's instructions. The library preparations were sequenced on an Illumina Hiseq platform, and 125 bp/150 bp paired-end reads were generated. Differential expression analysis (two replicates) was performed using the DESeq2 R package.

### Construction of subcutaneous in nude mice

Animal experiments were approved by the Ethics Committee of the Second Affiliated Hospital of Nantong University. Twelve BALB/c female nude mice aged 4 to 6 weeks (weighing approximately 20 g) were randomly divided into two groups. 1 x 10^6^ transfected OVCAR3 cells in 100 μL PBS were injected subcutaneously into the right flanks of mice. Tumor size was measured with a caliper every three days for six weeks. Tumor size was calculated as (length × width^2^)/2.

### Statistical analysis

Statistical analysis was performed with SPSS 22 and GraphPad 7.0 (GraphPad Software, La Jolla, CA, USA). Continuous data are expressed as mean ± standard deviation and were compared with the independent *t*-test. Categorical data are expressed as counts and were compared with Fisher's test. Comparisons of ≥3 groups were performed with one‐way analysis of variance (ANOVA). P < 0.05 was considered statistically significant.

## Results

### SNHG25 is up-regulated in EOC tissues

**Figure 1:**
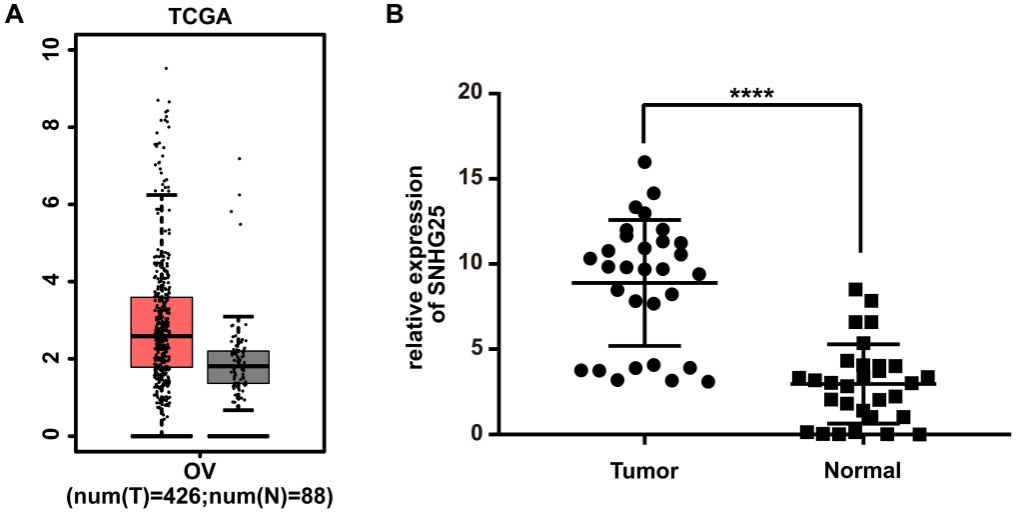
SNHG25 is up‐regulated in EOC. **A:** SNHG25 mRNA expression in tissues obtained from the TCGA database (n(tumor)=426, n(normal)=88); **B:** SNHG25 mRNA expression in tissues obtained from patients at the Second Affiliated Hospital of Nantong University (n(tumor)=30, n(normal)=30) (****P<0.0001).

Analyses using the TCGA database revealed that the expression of SNHG25 mRNA was significantly higher in EOC tissues compared to control tissues (Figure 1A). Consistent with these findings, the expression of SNHG25 mRNA was significantly higher in fresh frozen EOC tissues compared to control tissues obtained from patients at the Second Affiliated Hospital of Nantong University (Figure 1B). EOC tissues were stratified according to high or low expression of SNHG25 mRNA using median expression as the cut-off. High expression of SNHG25 mRNA was associated with International Federation of Gynecology and Obstetrics (FIGO) stage, histological grade (Table 1).

### Knockdown efficiency of SNHG25 was confirmed via qRT-PCR

**Figure 2:**
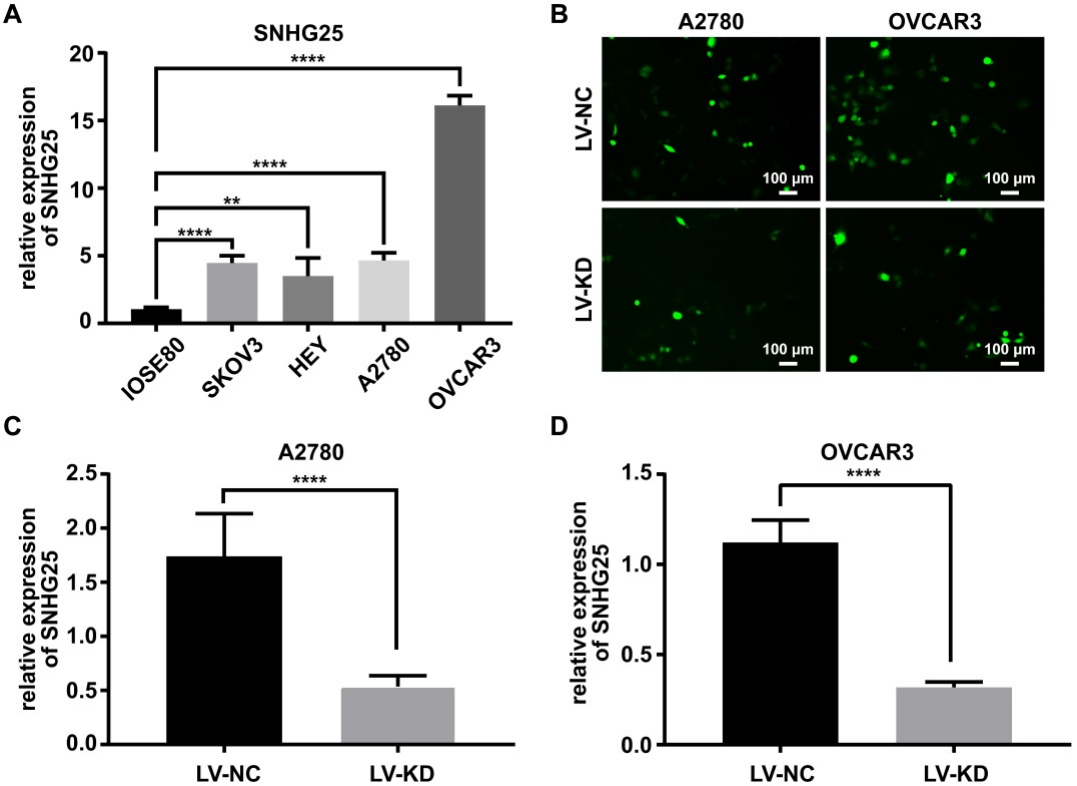
Knockdown efficiency of SNHG25 was confirmed via qRT-PCR. **A:** SNHG25 mRNA expression in four ovarian cancer cell lines (SKOV3, A2780 and HEY and OVCAR3) was significantly higher compared to normal ovarian surface epithelial cells (IOSE80); **B:** Fluorescence microscopy showing stable SNHG25 knockdown and control A2780 and OVCAR3 cell lines; **C-D:** SNHG25 knockdown efficiency was confirmed via qRT-PCR. (**P<0.01, ****P<0.0001).

The expression of SNHG25 mRNA was significantly higher in ovarian cancer cell lines (SKOV3, A2780 and HEY and OVCAR3) compared to normal ovarian surface epithelial cells (IOSE80) (Figure 2A). The expression of SNHG25 mRNA was highest in A2780 and OVCAR3 cells (Figure 2A); therefore, A2780 and OVCAR3 cells were chosen for the loss-of-function experiments. Stable SNHG25 knockdown and control A2780 and OVCAR3 cell lines were established (Figure 2B), and SNHG25 knockdown efficiency was confirmed via qRT-PCR (Figure 2C-D).

### Knockdown of SNHG25 inhibits proliferation of ovarian cancer cells *in vitro* and *vivo*

**Figure 3:**
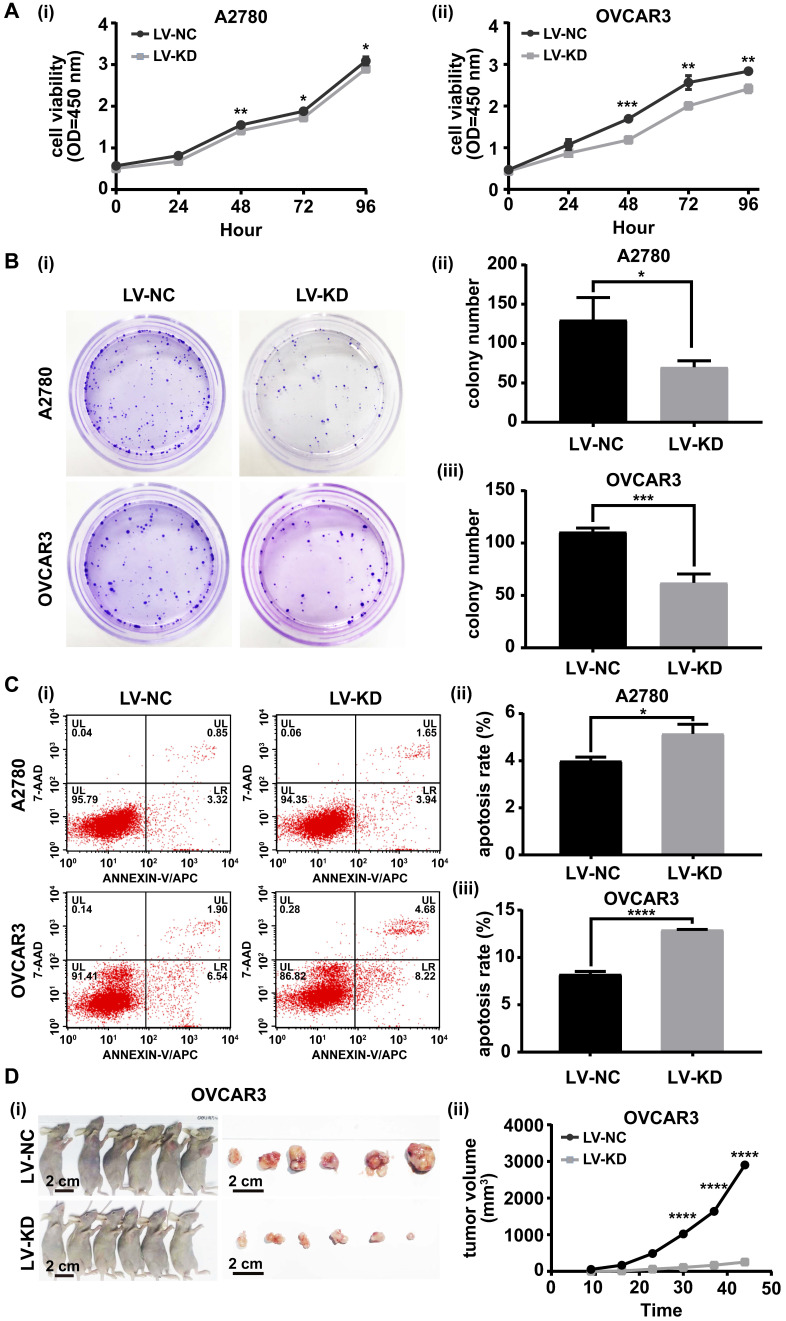
Knockdown of SNHG25 inhibits ovarian cancer cell proliferation *in vitro* and *in vivo*. **A(i-ii)-B(i-iii):** CCK-8 cell viability assay and colony formation assay showing decreased proliferation in SNHG25 knockdown compared to control A2780 and OVCAR3 cells (n(LV-NC)=3, n(LV-KD)=3); **C(i-iii):** Flow cytometry showing increased apoptosis in SNHG25 knockdown compared to control A2780 and OVCAR3 cells (n(LV-NC)=3, n(LV-KD)=3); **D(i-ii):** Downregulation of SNHG25 inhibited the growth (tumor volume) of subcutaneous xenografts in nude mice (n(LV-NC)=6, n(LV-KD)=6). (*P<0.05, **P<0.01, ***P<0.001, ****P <0.0001).

The role of SNHG25 in ovarian cancer cell proliferation was investigated with the CCK-8 cell viability assay, colony formation assay, flow cytometry and a nude mouse model bearing subcutaneous tumors. *In vitro*, the CCK-8 cell viability assay and colony formation assay indicated proliferation was significantly decreased (Figure 3 A (i-ii)-B (i-iii)) and flow cytometry revealed apoptosis was significantly increased in SNHG25 knockdown compared to control A2780 and OVCAR3 cells (Figure 3C (i-iii)). *In vivo*, downregulation of SNHG25 inhibited the growth (tumor volume) of subcutaneous xenografts in nude mice (Figure 3D (i-ii)).

### Knockdown of SNHG25 decreases ovarian cancer cell migration and invasion *in vitro*

**Figure 4:**
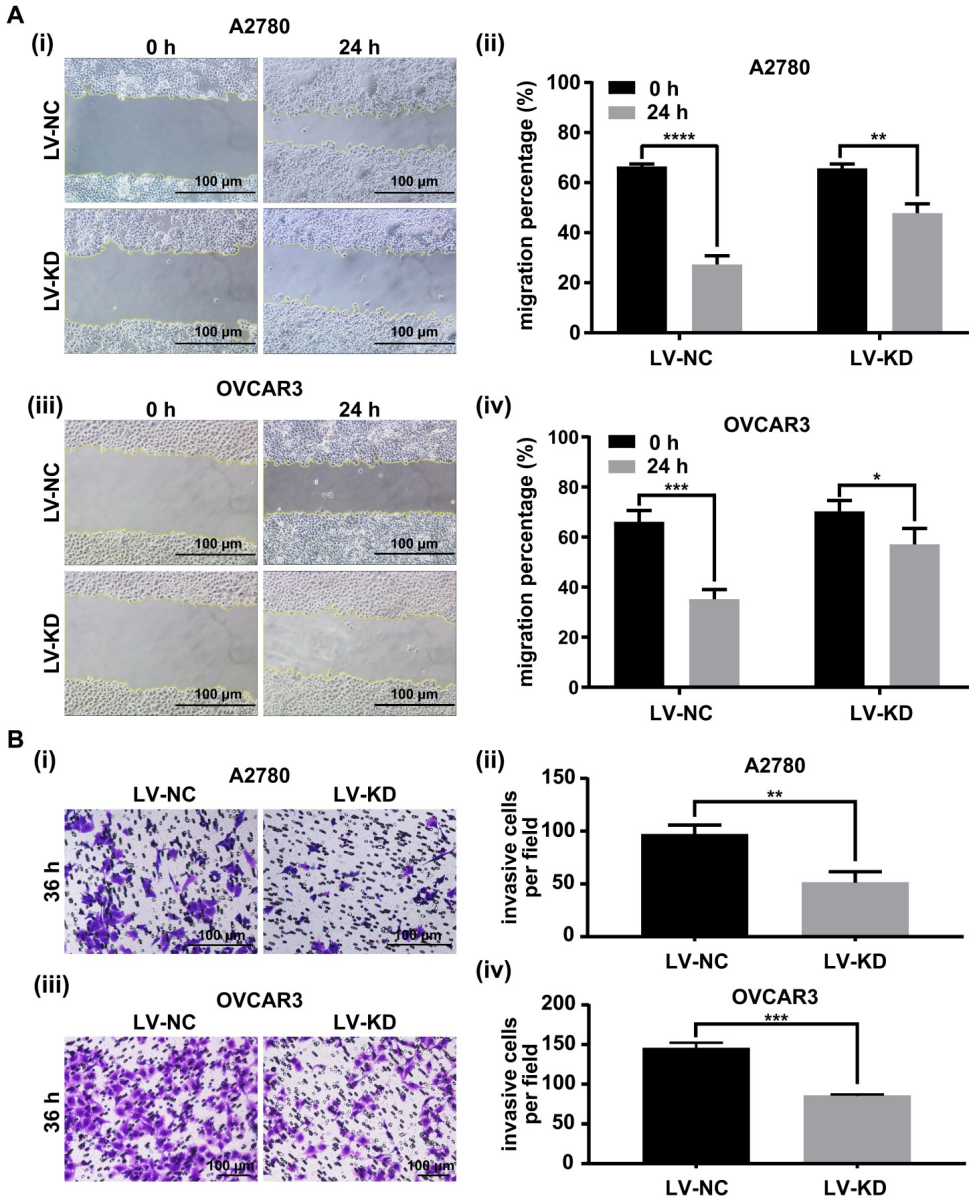
Knockdown of SNHG25 decreases ovarian cancer cell migration and invasion. **A(i-iv):** Scratch assay showing migration of ovarian cancer cells; **B(i-iv):** Transwell assay showing invasion of ovarian cancer cells. (n(LV-NC)=3, n(LV-KD)=3) (*P<0.05, **<0.01, ***P<0.001, ****P<0.0001).

The role of SNHG25 in ovarian cancer cell migration and invasion was investigated using the scratch assay and transwell cell migration assay. Migration (Figure 4A (i-iv)) and invasion (Figure 4B (i-iv)) were significantly decreased in SNHG25 knockdown compared to control A2780 and OVCAR3 cells.

### SNHG25 regulates the ovarian cancer progression by targeting COMP

**Figure 5:**
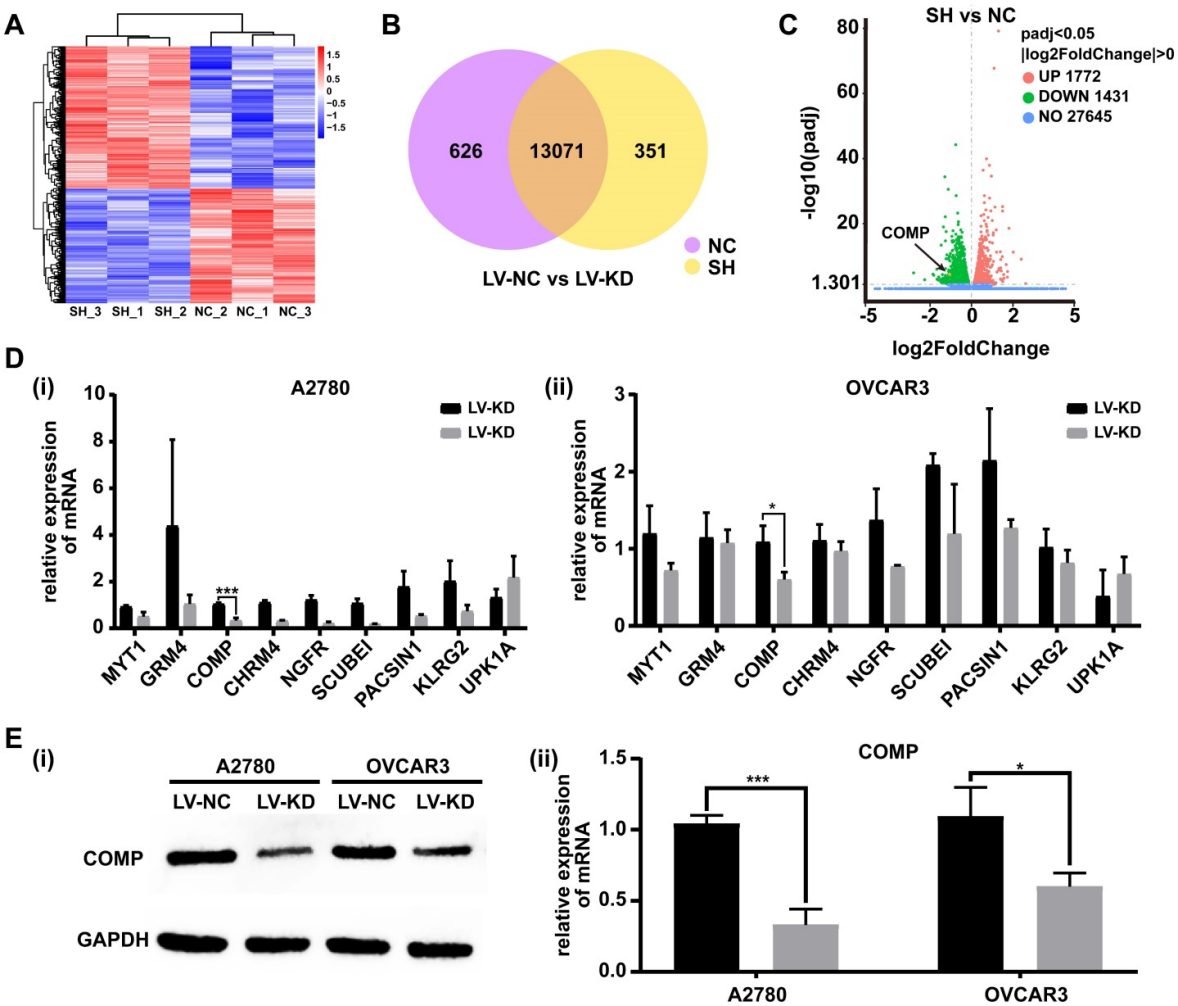
SNHG25 regulates ovarian cancer progression by targeting COMP. A-C: RNA sequencing data; **A:** Heatmap of the most variable genes with unsupervised hierarchical clustering of samples based on RNA sequencing data, SNHG25 knockdown (n=3) and control cells (n=3); **B:** Venn diagram comparing differentially expressed genes (SNHG25 knockdown (n=3) vs. control cells (n=3)); **C:** Volcano plot of differential gene expression in SNHG25 knockdown (n=3) vs. control cells (n=3). **D-E:** The top nine downregulated genes in SNHG25 knockdown (n=3) vs. control cells (n=3) included MYT1, GRM4, COMP, CHRM4, NGFR, SCUBE1, PACSIN1, KLRG2, and UPK1A. **D(i-ii):** mRNA expression measured by qRT-PCR. **E(i-ii):** COMP protein expression (n(LV-NC)=3, n(LV-KD)=3). (*P<0.05, ***P<0.001).

To explore the underlying molecular mechanisms involved in SNHG25-mediated proliferation, migration and invasion of ovarian cancer cells, transected cells were subjected to high-throughput sequencing to analyze differential gene expression. RNA-seq analysis identified differentially expressed genes in SNHG25 knockdown compared to control A2780 and OVCAR3 cells (Figure 5A-C). The top nine downregulated genes included MYT1, GRM4, COMP, CHRM4, NGFR, SCUBE1, PACSIN1, KLRG2, and UPK1A (Figure 5D (i-ii)). Among these, the expression of COMP (cartilage oligomeric matrix protein) mRNA was significantly decreased in SNHG25 knockdown compared to control A2780 and OVCAR3 cells. Consistent with this, there was a significant decrease in the expression of COMP protein in SNHG25 knockdown compared to control A2780 and OVCAR3 cells (Figure 5E (i-ii)).

## Discussion

There is an unmet clinical need to identify reliable biomarkers for the early detection of ovarian cancer. Currently, diagnosis of ovarian cancer is challenging due to the non-specific symptoms and lack of reliable early diagnostic tests. LncRNAs were originally considered as transcriptional “noise” or artifacts of RT-PCR 19. More recently, an increasing number of researchers have shown that lncRNAs play a pivotal role in the genesis and development of cancer. Evidence suggests that dysregulated lncRNAs participate in biological processes such as cell proliferation, migration, invasion and apoptosis in a variety of tumors 20-22, prompting lncRNAs to become a research hotspot 23.

The SNHG family may interact with tumor-related genes. Previous studies showed overexpression of SNHG12 was closely related to the development and poor prognosis of cervical cancer 24; SNHG6 was upregulated in colon and rectal adenocarcinoma, promoting tumorigenesis 25; SNHG8 regulated non-small cell lung cancer by influencing downstream effectors including CCND1 and CDK6^26^; and high SNHG20 expression was associated with shorter overall survival and SNHG20 was an independent risk factor for prognosis of serous EOC^27^.

Data extracted from the TCGA database imply a role for SNHG25, located in 17q23.3, in ovarian cancer. The present study revealed that SNHG25 was upregulated in EOC tissues and ovarian cancer cell lines, and SNHG25 knockdown decreased the proliferation, migration and invasion of ovarian cancer cells. These data indicate that SNHG25 might serve as an oncogene and promote EOC tumorigenesis.

COMP is a non-collagenous extracellular matrix protein expressed in cartilage, ligament, and tendon 28-30. COMP gene mutations can lead to pseudoachondroplasia and multiple epiphyseal dysplasia 31, 32. Recent studies have identified a vital role for COMP in carcinogenesis 33, 34, including an essential role for hepatic stellate cell-derived COMP overexpression in MEK/ERK and PI3K/AKT-mediated HCC progression 35, and a contribution to the development and metastasis of breast cancer^36^. However, the involvement of COMP in ovarian cancer remains to be elucidated. The present study demonstrated a significant decrease in the expression of COMP protein in SNHG25 knockdown compared to control ovarian cancer cells *in vitro*. These data suggest that SNHG25 may participate in EOC tumorigenesis by targeting COMP.

It is known that the SNHG family, including SNHG25, can participate in tumor progression. Some of the SNHG family members promote the tumor progression, while others may inhibit the progression of tumor. But SNHG family, like other lncRNAs, couldn't affect the proliferation, invasion and metastasis of tumor cells directly. They usually influence tumor progression by regulating targeting genes expression or signaling pathways. For example, SNHG4 facilitated the growth of osteosarcoma cells by regulating DOCK7 expression 16, and SNHG15 accelerated the development of hepatocellular carcinoma by targeting miR-490-3p/histone deacetylase2 axis 18. Furthermore, previous studies have found that different SNHG could promote the same tumor progression, such as both SNHG16 37 and SNHG20 27 promoting the progression of ovarian cancer. To our knowledge, regarding SNHG25, there is little knowledge about its roles in cancers. Our results showed that SNHG25 facilitated the proliferation, migration and invasion of ovarian cancer cells via regulating COMP expression. Our findings have enriched the roles and mechanisms of SNHG family in cancer biology.

This study was associated with several limitations. First, the sample size was small, increasing the risk of bias when extracting data. Second, prognosis was not explored. Third, further studies investigating the interaction between SNHG25 and COMP are required.

In conclusion, we present a novel role and possible mechanism of SNHG25 in EOC. Our results indicate that SNHG25 is highly expressed in EOC tissues, and is associated with FIGO stage, histological grade and CA125 level in patients with EOC. *In vitro* and *in vivo* findings demonstrated that SNHG25 promotes the proliferation, invasion and metastasis of ovarian cancer cells, potentially by regulating COMP.

## Figures and Tables

**Table 1:** Correlation between SNHG25 mRNA expression and clinicopathological parameters

Clinicopathological parameters	Cases (30)	LncRNA SNHG25 expression		P
		Low	High	
**Age(years)**				
≤55	9	5	4	0.418
>55	21	7	14	
**FIGO stage**				
Stage I-II	17	10	7	0.026
Stage III-IV	13	2	11	
**Histological grade**				
Grade 1-2	19	11	8	0.018
Grade 3	11	1	10	
**Pathological typing**				
Serous ovarian cancer	18	7	11	1.000
Mucinous ovarian cancer	12	5	7	

## Abbreviations

lncRNA: long non-coding RNA
EOC: epithelial ovarian cancer
SNHG: small nucleolar RNA host gene
COMP: cartilage oligomeric matrix protein
KD: knockdown
NC: negative control
RNA-seq: RNA-sequencing
qRT‐PCR: quantitative real-time polymerase chain reaction
